# Predictors of entrepreneurial intentions: The role of prior business experience, opportunity recognition, and entrepreneurial education

**DOI:** 10.3389/fpsyg.2022.882159

**Published:** 2022-11-03

**Authors:** Hongyun Tian, Shamim Akhtar, Naveed Akhtar Qureshi, Shuja Iqbal

**Affiliations:** ^1^School of Management, Jiangsu University, Zhenjiang, China; ^2^Management Sciences, Sukkur IBA University, Sukkur, Pakistan

**Keywords:** entrepreneurial intentions, prior business experience, opportunity recognition, entrepreneurial education, predictors of entrepreneurial intentions

## Abstract

Entrepreneurship and its influence on the development of the economy are significant in competitive global advancement. Entrepreneurs need entrepreneurial intentions to improve the commercial environment of the country. Therefore, studying entrepreneurial intentions’ influencing predictors is vital for business development. We collected data from small and medium-sized enterprises (SMEs) employees of the developing country and used partial least square structured equation modeling to analyze the proposed relationships. The results assist the literature extension and practically contribute to developing entrepreneurs’ intentions through education and opportunity recognition. The findings aid the institutions in improving course planning and establishing practical business setups. This study facilitates the government’s ideas of commencing entrepreneurial businesses through proper resource provisions for the entrepreneurs.

## Introduction

Economic development and innovativeness possess several advantages through enriching entrepreneurship outcomes, driving entrepreneurs’ substantial attention to launching a business (Asante and Affum Osei, 2019). A country’s economy intensely depends on the success, growth, and advancement of its prosperous business entities like small and medium-sized enterprises (SMEs) or large corporations (Chandra et al., 2020). However, concerning the economic contributions, including monetary benefits, job creation, dropping unemployment rate, poverty alleviation, industrial development, and export expansion, government places enormous attention on the development of SMEs (Fiseha and Oyelana, 2015). On global platforms, SMEs’ contribution reached 90% of total business development with the provision of almost 50% of job creation. SMEs contribute even higher to emerging economies by providing up to 90% of business with 70% of job establishment (Zafar and Mustafa, 2017). Subsequently, examining SMEs’ significance, development, improvement, innovation, and potential challenges is imperative. Here, it is crucial to notice that if SMEs are contributing comprehensively toward a country’s business, they need to be linked with entrepreneurial intentions.

There are several abilities of individuals that affect entrepreneurial intentions, such as personality traits (age and gender), knowledge (education and certifications), family background, family support, and prior work experiences (Duckworth et al., 2016; Yukongdi and Lopa, 2017). However, the entrepreneurship career does not need the aptitude to commence a particular start-up. It also focuses on “knowledge, resources, and creativity” (Chang and Chen, 2020; Paoloni et al., 2020). Therefore, this study aimed to investigate the significance of entrepreneurial education and prior business experience of the individuals (concerning knowledge) and how the opportunity for a creative entrepreneurship business has been recognized (concerning creativity) to boost entrepreneurial intentions.

Literature constantly analyzes different attitudes and predictors of entrepreneurship, entrepreneurial intentions, and decisions to become entrepreneurs (Grégoire et al., 2010). Despite the narration of several stories of entrepreneurial failures (Shu et al., 2018), the intention to start a business and become an entrepreneur increases (Palalić et al., 2017). Therefore, studying entrepreneurial intentions and their predictors is significant for literature and practical business situations. Considering diverse predictors of entrepreneurial intentions, studies admit that prior business experience can expedite the entrepreneurship career of the individuals (Quan, 2012). Individuals without previous business experience recognize different opportunities to develop intentions for entrepreneurship (Kuckertz and Wagner, 2010). Therefore, this study aimed to analyze the influence of prior business experience on entrepreneurial intentions.

Along with prior business experience, entrepreneurial education remains widely deliberated in the literature linked with entrepreneurial intentions (Liu et al., 2019). Education in terms of increasing the self-capability of the entrepreneurs influences entrepreneurial choices. Therefore, entrepreneurial education strongly predicts entrepreneurial intentions (Quan, 2012; Kim and Park, 2019) and contributes significantly to the country’s socio-economic development (Nguyen, 2018).

Effective recognition of opportunities for different business ideas also influences entrepreneurs’ intentions to take practical actions after the education individuals acquire (Bao et al., 2017). Recognizing the opportunity for a successful business venture requires understanding the importance of selecting a unique option from the ideas available *via* a diverse source of information (Kuratko and Morris, 2018). Entrepreneurs could use their knowledge and prior business experience to select a suitable option for entrepreneurship (Hassan et al., 2020). All individuals do not possess the ability and aspiration to commence business through strong entrepreneurial intentions; hence, there is a significant need to research the predictors of entrepreneurial intentions (Palalić et al., 2017). As an outcome, examining intentions could offer the policy makers the opportunity to understand the cognitive models of individuals thinking of indulging in entrepreneurial behavior more advantageous for opportunity recognition and policy-making than those who already imitated a business (Davidsson, 1995).

This study contributes to present research and theory by empirically examining the predictors of entrepreneurial intentions. The study addresses the gap by exploring the relationships between prior business experience and entrepreneurial intentions. Additionally, the study examined how entrepreneurial education and opportunity recognition influence entrepreneurial choices. Similarly, the study extends the investigation of entrepreneurial intentions by placing entrepreneurial education and opportunity recognition as mediators. Our research aimed to represent a significant level to the individuals in understanding the importance of the entrepreneurial intention’s predictors in the natural business environment.

## Literature review and hypothesis development

Entrepreneurial intentions are widely represented by the “Theory of Planned Behavior model” (Teixeira and Forte, 2017; Gorgievski et al., 2018; Al Jubari et al., 2019; Yuan et al., 2019; Alshebami et al., 2020). The theory explains that the attitude toward independent business start-ups describes the ability of an individual to give their intentions a practical shape (Nguyen, 2015). Therefore, the current study planned to investigate the practicality of entrepreneurial intentions through its predictors by explaining an individual’s ability (prior work experience, opportunity recognition, and entrepreneurial knowledge) in entrepreneurial business commencement.

Economists and departments of strategy development emphasize entrepreneurship and its predictors for boosting the country’s economic well-being (Doran et al., 2018; Urbano et al., 2020; Bazkiaei et al., 2021). The significance of entrepreneurial intentions (EI) in entrepreneurship procedure lies in eliminating unemployment rates for young entrepreneurs and individuals they hire in the business process (Pandit et al., 2018). For this reason, the research on entrepreneurial intentions always attracts scholars to translate entrepreneurial behaviors through the predictors of entrepreneurial intentions (Kautonen et al., 2015). Entrepreneurial intention refers to “the enthusiasm of an individual for completing an entrepreneurial activity to start a profitable business being self-employed.” Individuals bearing strong entrepreneurial intentions search for diverse information about the business and their capabilities to create the best plans for the future (Nguyen, 2018). Entrepreneurial intentions are “an attitude of passion for starting any business activity or constructing a profitable project” (Oluwafunmilayo et al., 2018). These intentions are significantly linked to the theory of planned behavior, as it states that intentions are the forces that derive any desired or planned behavior in an individual, as in entrepreneurs.

Prior business experience (PBE) influences the promotion of suitable start-ups in case of reduction of unemployment, especially in developing countries (Bignotti and Le Roux, 2020). Empirical studies can help understand the contribution of prior business experience in developing entrepreneurial intentions (Miralles et al., 2016). Additionally, previous family business experience directly affects the entrepreneurial choices of individuals to be self-employed rather than joining any institution for a job (Oluwafunmilayo et al., 2018). Prior business experience in entrepreneurial start-ups positively influences entrepreneurial intentions, increasing the self-employment of the individuals with the help of feasible business activity (Nguyen, 2018) significantly in young entrepreneurs (Chienwattanasook and Jermsittiparsert, 2019). However, research also claims that the entrepreneurs’ prior business experience negatively affects their entrepreneurial intentions because of prior awareness of the business, market, and industry (Kuckertz and Wagner, 2010). Prior work experience and entrepreneurial intentions need to be elaborated more in the literature (Yuan et al., 2019); hence, this research aimed to empirically analyze prior business experience as a predictor of entrepreneurial intentions. Prior business experience is a developed behavior that is an outcome of intentions toward being indulged in further entrepreneurial ventures using their past behavior. These behavioral intentions are a systematic process explained by theory of planned behavior. This study proposes the following relationships:

*H1*: Prior business experience positively and significantly affects entrepreneurial intentions.

*H2*:Prior business experience positively and significantly affects entrepreneurial education.

Entrepreneurial education is considered a strong competency that entrepreneurs with previous business experience need to attain for the growth of the business (Bauman and Lucy, 2019). Prior business exposure positively influences entrepreneurial education and develops the intentions of the entrepreneurs to receive entrepreneurial education (Ramayah et al., 2012). However, there is a significant need to provide empirical findings on receiving an education without prior business experience (Ugwuozor, 2020). Hence the study proposed analyzing the relationship between prior business experience and entrepreneurial education. Additionally, individuals develop a strong attitude toward entrepreneurial intentions with the help of entrepreneurial education (Bazan et al., 2019), as education is one of the strong motivators for an entrepreneurship career (Urbano et al., 2020). Likewise, education is also an outcome of a significantly planned behavior because individuals intend a suitable education, apply for it, and get (receive) their desired education.

Moreover, education also boosts the intention of students to become an entrepreneur (Anwar and Saleem, 2018). Therefore, advancement in the entrepreneurial education and entrepreneurial intentions of the individuals should be increased. Several universities offer different entrepreneurship subjects to improve their education, skills, and passions to become entrepreneurs (Palalić et al., 2017). Entrepreneurial education describes the totality of “training, skills, knowledge and inspiring accomplishments attained from an educational institute that enable the students to achieve entrepreneurial skills to hunt entrepreneurial business effectively” (Bazkiaei et al., 2020). In other words, entrepreneurial education is the training plan aimed increasing the skills, ethical conducts and knowledge essential for student aiming to become an entrepreneur (Doan and Phan, 2020).

Entrepreneurial education positively affected entrepreneurial intentions as the entrepreneurial prior business experience became mainstream entrepreneurial education (Nabi et al., 2017). Entrepreneurship education increases entrepreneurial intentions because the individual possesses theoretical knowledge about the business, market, and industry (Anwar et al., 2020). The research investigated the effect of entrepreneurial education on entrepreneurial intentions (positive results; Hassan et al., 2020), which turns entrepreneurial intentions into actual business activity (Raza et al., 2018). Higher levels of entrepreneurial education yield a higher probability of being self-employed (Nabi et al., 2017). Education systems provide individuals with the best opportunities to attain diverse knowledge about business start-ups (Hameed and Irfan, 2019). Different programs and courses offered by institutions increase the skills of individuals to perform better in the entrepreneurship process (idea to business setup procedure; Shahverdi et al., 2018).

The economy develops under the efforts and success of entrepreneurs who bring innovations and improvement to businesses; hence education and research institutes (all over the world) offer entrepreneurial education programs to meet economic development goals (Nabi et al., 2018). The economic benefits of entrepreneurial education are considered significantly significant with the focus on the heterogeneity of the entrepreneurs’ educational background (“subjective development, professed aptitude, and social influence; Teixeira and Forte, 2017). Therefore entrepreneurs’ education attained importance in entrepreneurship and entrepreneurial intentions (Sun et al., 2017).

Research claims that the education of entrepreneurship (as mediator) enhances the capability of entrepreneurs and other individuals to build solid entrepreneurial intentions assisted by self-employment (Neergaard and Christensen, 2017). Therefore entrepreneurial education helps in career development by influencing the decisions of individuals to become successful entrepreneurs (Bazkiaei et al., 2020). Education is an experimental mediating feature with experiences gained from prior business activities (Kassean et al., 2015). Individuals who believe “learning with doing” give more output in entrepreneurial intentions through business experiences (Nabi et al., 2017). Therefore, the study aimed to investigate the mediating effect of entrepreneurial education on the relationship of prior expertise and entrepreneurial intentions. This study has found that entrepreneurial education has positive and significant links with prior business experience, showing positive relations (Chienwattanasook and Jermsittiparsert, 2019). Entrepreneurial education also positively affects entrepreneurial intentions (Hassan et al., 2020). Likewise, prior business experience also directly affects the entrepreneurial intentions (Doran et al., 2018). Considering these direct links, this study proposes that prior business experience affects entrepreneurial intentions when mediated by entrepreneurial education. This notion develops a sense that individuals having prior business experience will develop strongly entrepreneurial intentions when they will enhances their understanding and knowledge of entrepreneurship through formal education or business-oriented degree programs, diploma, or certifications. In addition, as per the theory of planned behavior, intentions are outcomes of the planned behavior such as entrepreneurial education and prior business experience. Thus, the current study proposes the following relationships:

*H3*: Entrepreneurial education positively and significantly impacts their entrepreneurial intentions.

*H4*: Entrepreneurial education mediates the relationship between prior business experience and entrepreneurial intentions.

One of the influential factors in pursuing an entrepreneurial career is the aptitude to recognize suitable entrepreneurship opportunities (Hanohov and Baldacchino, 2018). Opportunity recognition refers to the “recognition of a plan/chance about a suitable arrangement of knowledge, skills, and resources that can lead toward starting a successful business with profit generation ability” (Kuckertz et al., 2017). Research focuses on the quality and necessity of the entrepreneurial opportunity recognized rather than “how” it has been explored by the entrepreneurs (Asante and Affum Osei, 2019). Opportunity recognition defines “the ability of the individual to identify, distinguish or build ideas and models about a particular business activity (in entrepreneurial setups)” (Hassan et al., 2020). Opportunity recognition serves as a critical phenomenon of entrepreneurial intentions presenting a choice of self-employment (Manesh and Rialp-Criado, 2019) that gives meaning to the actions that discover, evaluate and exploit diverse business opportunities (Bao et al., 2017). The necessity of opportunity recognition is a significant variable because of the consensus of the scholar that entrepreneurs cannot pursue entrepreneurial careers without exploring opportunities for diverse businesses (Asante and Affum Osei, 2019). Recognition of entrepreneurial opportunities by individuals became an essential element of entrepreneurial intentions (Vuorio et al., 2018). Prior business experience has been stated as one the primary elements concerning entrepreneurial opportunity recognition. It is suggested that combined understanding and experiences, specifically past career perspectives offers the conditions for developing a more confident decision of entrepreneurial opportunities. Essentially, it is suggested that the attainment of understanding and experiences supports the opportunity recognition and linked required skills (Baeta and Andreassi, 2021). This study proposes that:

*H5*: Prior business experience positively and significantly affects opportunity recognition.

Successful entrepreneurs must recognize many opportunities to develop solid entrepreneurial intentions (Hanohov and Baldacchino, 2018). In opportunity recognition, entrepreneurs search for a good idea for a specific business using several information sources. Executing these ideas into business activity (Hassan et al., 2020). Three essential factors assist the recognition of entrepreneurship opportunities. First, “engaging in active research” enables entrepreneurs to analyze the emergence and profitability of particular opportunities. The second factor includes the customers served through a particular product or service. A third factor focuses on the prior business knowledge in the industry (Dahalan et al., 2015). Hence this research chooses the “prior business experience” to investigate in recognizing the entrepreneurial opportunity. Identifying entrepreneurial opportunities in launching a business can provide an essential understanding of entrepreneurial intentions in the entrepreneurship progression (Shu et al., 2018). Opportunity recognition also needs to discuss the risks when entrepreneurs unfold the information about different ideas to exploit the success of business plans. Hence opportunity recognition develops viable business plans to enhance entrepreneurial intentions toward potential growth (Shamsudeen et al., 2017) by acting as a mediator (Bao et al., 2017; Asante and Affum Osei, 2019).

This study proposes the mediating mechanism of opportunity recognition; it is evident that there are significant effects of prior business experience on opportunity recognition (Asante and Affum Osei, 2019) and entrepreneurial intentions (Yuan et al., 2019). Likewise, opportunity recognition also directly influences entrepreneurial intentions (Hassan et al., 2020). This paper states that the individuals having prior business experience have some abilities and skills to recognize opportunities for conducting business and this it strengthens their intentions to do so. In the same vein, the theory of planned behavior states that intentions and beliefs could be learnable but not inborn (Krueger et al., 1994). It is argued in light of the theory that personal variations such as past work experience, understanding, and ability of opportunity recognition could influence the change of intentions (Ajzen et al., 2009). As per the theory of planned behavior, individual behavior is an outcome of the intention to start a business and earn experience, allowing an entrepreneur to recognize the possible opportunities in the market. Such intentions and behavior are vital for entrepreneurs and the business’ success. According to the authors’ knowledge, there is scarcely evidence of the relationship proposed below in the context of Pakistan; therefore, the study proposed the following hypothesis.

*H6*: Business Opportunity recognition positively and significantly impacts entrepreneurial intentions.

*H7*: Business Opportunity recognition mediates the relationship between prior business experience and entrepreneurial intentions.

**Figure 1:**
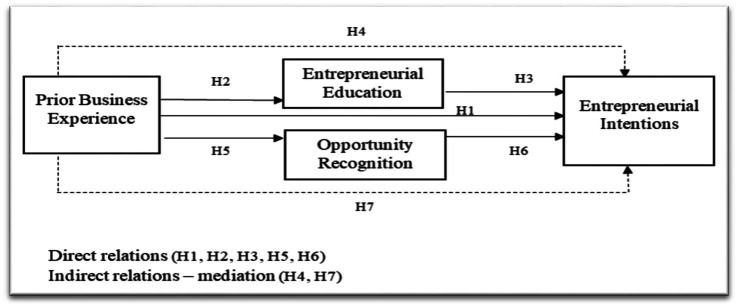
Research framework.

The above research framework details the investigated model of the study (Figure 1).

## Research methodology

The proposed hypotheses of the study were tested by conducting a survey in-lined with the former research regarding entrepreneurial intentions (Palalić et al., 2017; Nguyen, 2018; Oluwafunmilayo et al., 2018; Asante and Affum Osei, 2019; Alshebami et al., 2020; Bazkiaei et al., 2020). Data is collected from entrepreneurs with prior business experience and an entrepreneurship education from an institute (business degree, entrepreneurship education, business certification, and others). To ensure that the study respondents had prior business experience the questionnaire included a statement to enquire a ‘yes’ or ‘no’ response (and it was advised that only the respondents having the prior business experience should proceed their response). Once the data was collected, it was found that around 2–3 respondents did not had prior work experience and those observations were excluded from the data set. In order to collect data, a well-prepared questionnaire was developed and self-administered to SMEs of Pakistan. The web-based questionnaire was dispersed to the entrepreneurs of SMEs with the help of a link through different social media platforms, including email and WhatsApp, after confirming their online existence. The questionnaire contained the consent statement ensuring confidentially to the participants regarding privacy concerns. The questionnaire covered the aspects of selected variables as Prior business experience (PBE), Entrepreneurial Education (EE), Opportunity Recognition (OR), and Entrepreneurial Intentions (EI). The study followed the random sampling technique, in line with the previous research (Palalić et al., 2017; Alshebami et al., 2020).

### Study measure

This study measured the variable using the scales developed by previous studies. For instance, the prior business experience was measured by adopting three items from the study of Bignotti and Le Roux (2020). Opportunity recognition was measured with five items adopted from Hassan et al. (2020); Liñán and Chen (2009). Likewise, entrepreneurial education and intentions were measure with five items each adopted from the study of Hassan et al. (2020). These scales are listed in Table 1. All the variables were measured using a five-point Likert scale as used in these past studies.

**Table 1:** Items of measurement.

Items of measurement	Source
Prior Business experience	Bignotti and Le Roux (2020)
“I have worked in my parents’ business” for entrepreneurial early childhood experiences.” “I have attempted to start a business” for prior start-up experiences.” “I have done a holiday job” for work experience.”	
Opportunity recognition	Hassan et al. (2020)
“I see many opportunities to start and grow a business.” “Finding potential venture opportunities is easy for me.” “In general, there are many opportunities for new product innovation.” “I have a special sense of new venture ideas.” “During my routine day-to-day activities, I see potential new venture ideas.”	
Entrepreneurship education	Hassan et al. (2020) and Liñán and Chen (2009)
“Knowledge about the entrepreneurial environment” “Greater recognition of the entrepreneur’s figure” “The preference to be an entrepreneur” “The necessary abilities to be an entrepreneur” “The intention to be an entrepreneur”	
Entrepreneurial intention	Hassan et al. (2020)
“I am ready to make anything to be an entrepreneur.” “My professional goal is to become an entrepreneur.” “I will make every effort to start and run my own firm.” “I am determined to create a firm in the future.” “I have very seriously thought of starting a firm.” “I have got the firm intention to start a firm someday.”	

After data collection, the filled questionnaires were analyzed using SmartPLS software. Data collection tool must show consistency with the developed research model (Rafiq and Chin, 2019); therefore study chose SmartPLS as it is widely used in business research analysis (Kwamega et al., 2019; Cheah et al., 2020; Thaker et al., 2020; Tian et al., 2021). A total of 700 questionnaires were administered. However, correct responses are collected from 341 questionnaires provided by the entrepreneurs (48.71% response rate), depicting a reasonable sample size for analyzing the variables (Roscoe, 1975; Wolf et al., 2013). Before proceeding to the measurement model and hypotheses testing the distribution of the measures was assessed. In this regard, the mean, standard deviation, skewness, and kurtosis were examined. Table 2 shows the descriptive statistics of 341 observations showing the accumulative number of non-missing values. Mean shows the center of the sample observations, like for PBE1 = 3.818. The spread in data is shown by the values of standard deviation, the higher values show the greater spread. Skewness exhibits the patterns (non-symmetrical) in the data. When the value of the item reaches zero (0), the data turns more symmetrical. While the negative values exhibits left and the positive values show right-skewed data. The sample in this study shows the left-skewed data. Moreover, kurtosis exhibits the variation among the tails and peaks of a distribution. A zero value for kurtosis shows complete normal distribution in the data for example, EI3 = 0.630. In this study the kurtosis shows that the distribution has lighter tails as compared to normal distribution.

**Table 2:** Descriptive statistics of measures.

	Mean	STDV	Kurtosis	Skewness
PBE1	3.818	1.02	1.947	−1.462
PBE2	3.933	0.977	1.141	−1.153
PBE3	4.053	1.037	1.241	−1.245
EE1	3.818	1.02	1.947	−1.462
EE2	3.933	0.977	1.141	−1.153
EE3	4.053	1.037	1.241	−1.245
EE4	3.953	0.98	0.949	−1.09
EE5	3.877	0.976	1.304	−1.157
OR1	3.24	1.427	−1.291	−0.381
OR2	3.384	1.454	−1.373	−0.333
OR3	3.196	1.512	−1.458	−0.296
OR4	3.191	1.402	−1.344	−0.227
OR5	3.214	1.359	−1.179	−0.379
EI1	3.361	1.343	−1.159	−0.409
EI2	3.522	1.356	−0.887	−0.66
EI3	3.44	1.412	−1.163	−0.479
EI4	3.355	1.522	−1.371	−0.429
EI5	3.921	0.982	0.63	−0.979
EI6	3.877	0.958	1.171	−1.079

## Results

### Measurement model

**Figure. 2:**
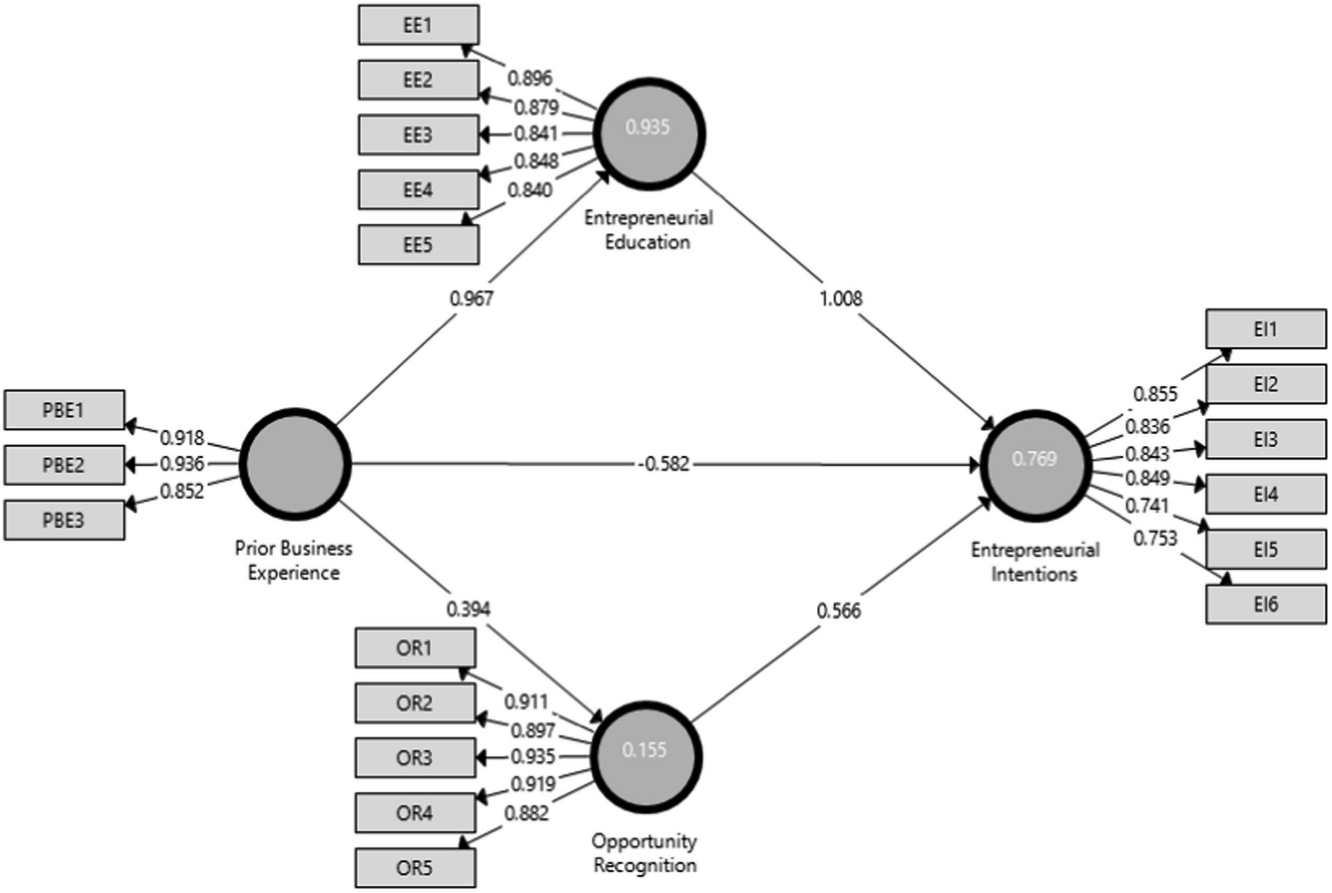
Model outer loadings.

Table 2 and Figure 2 display the outer loadings of the model items along with the sound, consistent reliability (CR) values between 0.922–0.960 that fall in the threshold range (above or equal to 0.7; Hair et al., 2016; De Souzabido and Da Silva, 2019). The Cronbach alpha (CA) values determine the reliability of the questionnaire items. Results show significant validity measures of CA, 0.913 for EE, 0.898 for EI, 0.947 for OR, and 0.885 for PBE. Values of Average Variance Extract (AVE) meet the criteria of at least 0.5 (Ab Hamid et al., 2017; Lee and Chen, 2018). The model analysis revealed the AVE values 0.741 for EE, 0.663 for EI, 0.826 for OR, and 0.815 for prior business experience. Results showed significant values for discriminant validity with positive correlation among the variables (values greater than 0.5; Table 3). The values of Heterotrait-Monotrait Ratio of Correlations (HTMT; Table 4) lie between 0.429–0.843, meeting the threshold significant value (should be less than 0.90/0.85; Sari, 2020; Iqbal et al., 2021).

**Table 3:** Measurement model.

Constructs	Items	Loadings	Weights	CA	CR	AVE	VIF
Entrepreneurial education	EE1	0.896	0.250	0.913	0.935	0.741	1.231
	EE2	0.879	0.243				
	EE3	0.841	0.228				
	EE4	0.848	0.220				
	EE5	0.840	0.219				
Entrepreneurial intentions	EI1	0.855	0.192	0.898	0.922	0.663	1.201
	EI2	0.836	0.190				
	EI3	0.843	0.197				
	EI4	0.849	0.196				
	EI5	0.741	0.230				
	EI6	0.753	0.231				
Opportunity recognition	OR1	0.911	0.224	0.947	0.960	0.826	1.234
	OR2	0.897	0.216				
	OR3	0.935	0.219				
	OR4	0.919	0.218				
	OR5	0.882	0.224				
Prior business experience	PBE1	0.918	0.389	0.885	0.929	0.815	1.000
	PBE2	0.936	0.364				
	PBE3	0.852	0.355				

AVE, average variance extracted; CA, Cronbach’s alpha; CR, composite reliability.

**Table 4:** Discriminant validity (latent variable correlation and square root of AVE).

	Entrepreneurial education	Entrepreneurial intentions	Opportunity recognition	Prior business experience
Entrepreneurial education	*0.967*			
Entrepreneurial intentions	0.688	*0.814*		
Opportunity recognition	0.428	0.768	*0.909*	
Prior business experience	0.861	0.616	0.394	*0.903*

The italic values shows the square root of AVE confirming discriminant validity.

This study assessed common method bias and collinearity issues with Variance Inflation Factor (VIF), which is considered the reciprocal of tolerance. The results show that no values were more significant than 3.3. Thus the study is bias-free (Hair et al., 2016).

### Structural model

Q^2^ determines the predictive relevance of the model. The predictive relevance of the model needs to be more than zero according to the threshold values (Ringle et al., 2012; Silaparasetti et al., 2017); hence the analysis showed significant values. The Standardized Root Mean Square Residual (SRMR) value is less than 0.08, according to (Munoz et al., 2020; Pavlov et al., 2021). Therefore, the result of this study about SRMR is significant. R^2^ (R Square) explains the impact of independent variable/s on dependent variable/s (Anondho et al., 2019). The threshold value interpret 0.75 = substantial, 0.5 = moderate, and 0.25 = weak impact (Jeo et al., 2011). Table 5 indicates the R^2^ value of the model.

**Table 5:** HTMT (Heterotrait-Monotrait ratio).

	Entrepreneurial education	Entrepreneurial intentions	Opportunity recognition
Entrepreneurial intentions	0.738		
Opportunity recognition	0.461	0.843	
Prior business experience	0.841	0.670	0.429

The value of R^2^ (Entrepreneurial Education-0.935, Entrepreneurial Intentions-0.769, and Opportunity Recognition-0.115) explains that 93.5%, 76.9%, and 11.5% impact is experiential in entrepreneurial education, entrepreneurial intentions, and opportunity recognition by prior business experience, respectively, expressing effect occurs between the variables.

### Structural equation modeling

**Figure. 3:**
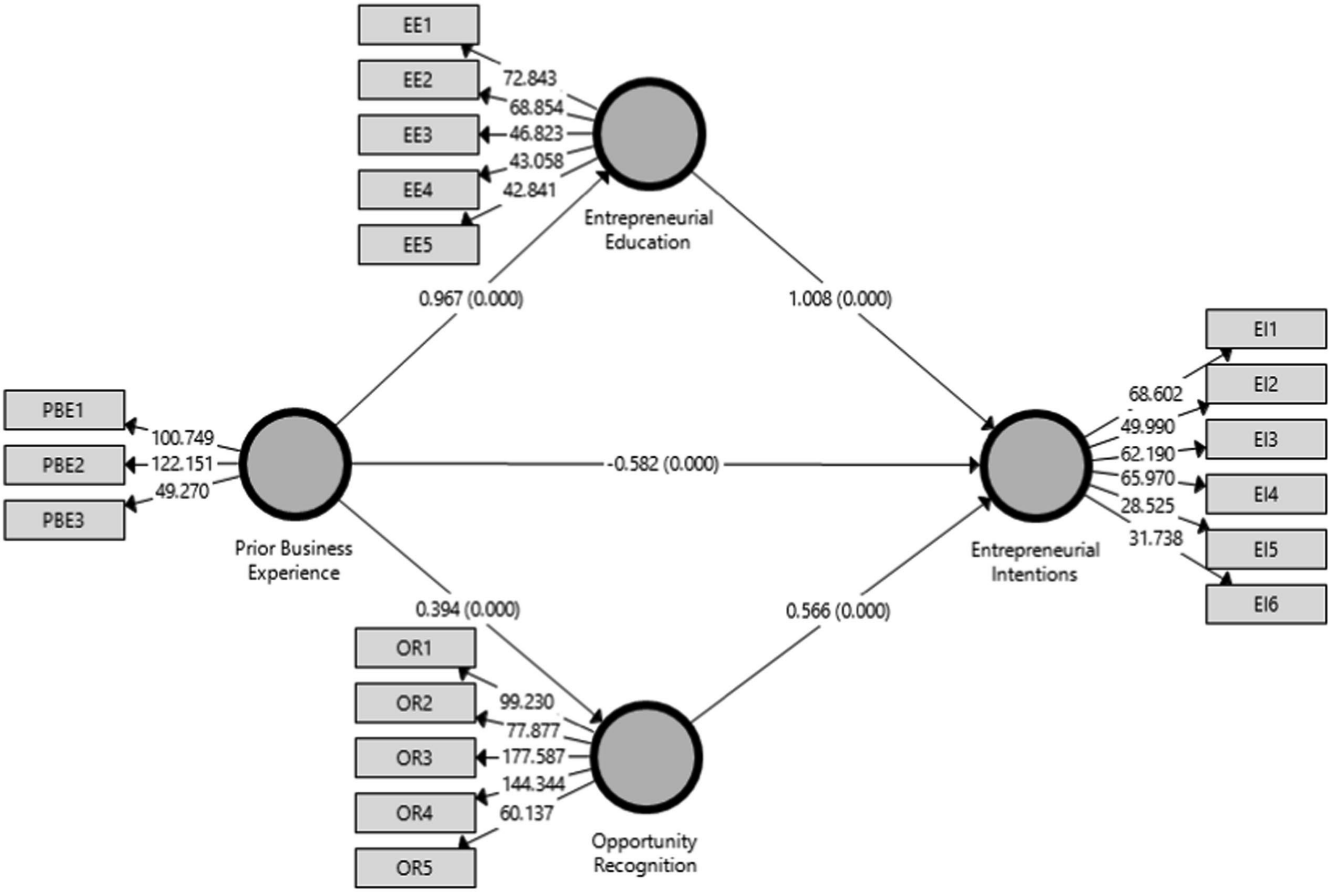
PLS-SEM model.

The presented theoretical model is analyzed using Smart PLS—Structural Equation Modeling (SEM). The analysis show that prior business experience has positive and significant impact on entrepreneurial intentions (β = 0.616, *t* = 15.523, *p* < 0.000). Similarly, the direct impact of prior business experience on entrepreneurial education (β = 0.967, *t* = 283.475, *p* < 0.000) and direct impact of prior business experience on opportunity recognition (β = 0.394, *t* = 9.515, *p* < 0.000) show significant and positive impacts. Moreover, the direct effect of entrepreneurial education (β = 1.008, *t* = 9.023, *p* < 0.000) and opportunity recognition (β = 0.566, *t* = 15.113, *p* < 0.000) on entrepreneurial intentions is also positive and significant. Entrepreneurial education and opportunity recognition both as mediator show positive and significant impact (β = 0.975, *t* = 9.048, *p* < 0.000), (β = 0.223, *t* = 8.164, *p* < 0.000) between the relationship of prior business experience and entrepreneurial intentions (Table 6; Figure 3).

**Table 6:** Model strengths.

Construct	Effects size			Coefficient of determination (R2)		SRMR
	SSO	SSE	Q2 (=1-SSE/SSO)	R^2^	Adj. R^2^	
Entrepreneurial education	1,705	601.365	0.647	0.935	0.935	0.067
Entrepreneurial intentions	2,046	1088.443	0.468	0.769	0.767	
Opportunity recognition	1,705	1501.48	0.119	0.155	0.153	
Prior Business experience	1,023	1023				

Q^2^, predictive relevance; SRMR, standardized root mean square; R^2^, determination of coefficient.

All the hypothesis results have consistency with previous research (Ramayah et al., 2012; Dahalan et al., 2015; Anwar and Saleem, 2018; Vuorio et al., 2018; Manesh and Rialp-Criado, 2019; Bazkiaei et al., 2020; Bignotti and Le Roux, 2020; Ugwuozor, 2020; Table 7).

**Table 7:** Hypothesis constructs.

Effects	Relationships	Beta	Mean	(STDEV)	*t*-value	Decision
**Direct**						
	Entrepreneurial education entrepreneurial intentions	1.008	1.008	0.112	9.023^*^	Supported
	Opportunity recognition entrepreneurial intentions	0.566	0.566	0.037	15.113^*^	Supported
	Prior business experience entrepreneurial education	0.967	0.967	0.003	283.475^*^	Supported
	Prior business experience entrepreneurial intentions	0.616	0.615	0.04	15.523^*^	Supported
	Prior business experience opportunity recognition	0.394	0.395	0.041	9.515^*^	Supported
**Indirect or mediating**						
	Prior business experience entrepreneurial education entrepreneurial intentions	0.975	0.975	0.108	9.048^*^	Supported
	Prior business experience opportunity recognition entrepreneurial intentions	0.223	0.224	0.027	8.164^*^	Supported

The * shows that all the values were within the suggested confidence interval of 95% (*p*-value).

## Conclusion and discussion

### Discussion

The study contributes to the evolving stream of literature that assists in integrating knowledge about positive entrepreneurial intentions and their predictors. The study contributes different impacts and relationships of prior business experience with entrepreneurial education, opportunity recognition, and entrepreneurial intentions in the literature. Entrepreneurs with previous business experience can attempt entrepreneurial start-up-activities (Nguyen, 2018). Entrepreneurs with business experience can quickly find opportunities in the market for a new start-up or previous innovative activities. Entrepreneurs always stay ahead to search for potential opportunities besides the daily routine of life with the confidence to start and grow a business (Osterwalder et al., 2020). Believe in their abilities and the success of enterprises providing innovative products by recognizing various opportunities in the market demanded by the customers. Individuals prefer to be entrepreneurs when they learn different courses about entrepreneurship as it builds the foundation of knowledge about the business environment and recognizes opportunities (Rudhumbu et al., 2020). Additionally, deep learning of entrepreneurship subjects reveals particular abilities that make an individual an entrepreneur (Bae et al., 2014), hence developing solid entrepreneurial intentions. Hence, this empirical study can help understand the contribution of prior business experience in developing entrepreneurial intentions.

The study validates all constructed hypotheses. Results show that prior business experiences of the entrepreneurs positively impact their entrepreneurial intentions (H1), similar to previous studies (Miralles et al., 2016; Chienwattanasook and Jermsittiparsert, 2019; Bignotti and Le Roux, 2020). The supported hypothesis indicates that the experience of the entrepreneurs in handling a business motivates them to start more businesses. They possess strong intentions to work for entrepreneurial businesses. Empirical values indicate that prior business experience inserts a positive and significant impact on entrepreneurial education and opportunity recognition (H2, H5), showing consistency with (Ramayah et al., 2012; Dahalan et al., 2015; Shu et al., 2018; Ugwuozor, 2020). Entrepreneurs prefer to attain education to help create a successful business activity. Moreover, individuals with previous business know-how can search for new opportunities that enhance their opportunity recognition abilities. In return, entrepreneurial education entrepreneurs seem more motivated to commence. With the enhanced capability of opportunity recognition, entrepreneurs believe themselves more to increase entrepreneurial intentions. The results support the proposed hypothesis (H3, H6), similar to previous research (Nabi et al., 2017; Anwar and Saleem, 2018; Raza et al., 2018; Vuorio et al., 2018; Asante and Affum Osei, 2019; Manesh and Rialp-Criado, 2019). Additionally, the study observes that opportunity recognition and entrepreneurial education mediate the relationship between prior business experience and entrepreneurial intentions (H4, H7). The results show consistency with a scholar (Kassean et al., 2015; Bao et al., 2017; Shamsudeen et al., 2017; Bazkiaei et al., 2020). Entrepreneurs with prior business experience depict higher entrepreneurial intentions when they gain theoretical knowledge (about entrepreneurship) from the educational institute and attain good opportunities from the market.

The study establishes profound foundations for entrepreneurs to use their prior business experience and skills in attaining new opportunities. Data reveals that entrepreneurs who already have previous investment experience can easily find several opportunities for new ventures (Bignotti and Le Roux, 2020). Therefore, entrepreneurs should observe new business perspectives in daily routine activities, either to start their own business or to recommend others for social betterment. However, education plays a crucial role in boosting entrepreneurial intentions (Rudhumbu et al., 2020). Respondents agree that a degree or certification in business and management is necessary to become an entrepreneur as it enhances intentional abilities. Hence institutions and entrepreneurs should develop a link among themselves to educate the individuals on practical business activities under strong entrepreneurial aptitude and intentions.

### Conclusion

#### Practical implications

Understanding and practicing entrepreneurship is significant for developing the economy and societal well-being. Prior work experience builds confidence in the entrepreneurs to invest in different business activities by searching for suitable opportunities. Therefore, government, provincial authorities, and investors can support the individuals in commencing business by providing opportunities and financial support. With this support, Entrepreneurs will work with extra supportive intentions toward starting a business that will ultimately boost the country’s economy and entrepreneurship advancement by increasing gross domestic product (GDP). Entrepreneurial solid intentions and predictors can assist in business activities and entrepreneurial careers. The study of entrepreneurial intentions can help the economy reduce the unemployment rate.

Additionally, it can provide a wide range of opportunities and learning to boost the entrepreneurial career by using entrepreneurial education. This study offers practical implications to educational institutes as individuals with entrepreneurship education hold strong intentions to start a business. Therefore, institutions should insert efforts to provide advanced knowledge of entrepreneurship in subjects and curricular activities. Academic institutions can focus on providing possible situations representing the practical shape of theoretical knowledge to boost students’ entrepreneurial intentions. There is a need to increase the bond between theory and practice regarding entrepreneurial concepts and actual business start-ups. Hence, universities, colleges, and other business education institutes will ensure the proper training of the teachers and professors that can *impress and inspire* (increasing entrepreneurial intentions) the generations to become future entrepreneurs.

In parallel with institutions’ efforts for entrepreneurial education development, entrepreneurs should also create awareness about the advantages of education to individuals. Concerning the purpose, entrepreneurs and institutions should collaborate to arrange beneficial workshops and seminars (exemplifying real success stories) that can boost the intentions of individuals to start a business.

#### Limitations and future research directions

The study is not free from limitations that can be addressed by future research. Firstly, the study investigated only single dimensions of variables; however, multidimensional variables can predict new theoretical and practical perspectives. Secondly, the study is conducted on the small and medium enterprises of Pakistan, considered a developing nation, whereas the selected variables can be extended to examine other developing nations as well. Considering the variables, Future research can target comparative studies to analyze entrepreneurial intentions in different individuals. For example, the analysis can investigate the level of intentions between the entrepreneurs with and without entrepreneurial education. Similarly, the difference between entrepreneurs with prior experience and those new to the business can be analyzed. Moreover, the dimensions of prior business experience can be explored in future research, such as related or unrelated prior business experience while starting a new business.

## Data availability statement

The raw data supporting the conclusions of this article will be made available by the authors, without undue reservation.

## Ethics statement

The study was conducted according to the guidelines of the Declaration of Helsinki. The Jiangsu University review board exempted the research for ethical approval, as it is a survey-based study. The study obtained the employees’ consent working in the SMEs, and they filled the questionnaires willingly. The patients/participants provided their written informed consent to participate in this study.

## Author contributions

All authors listed have made a substantial, direct, and intellectual contribution to the work and approved it for publication.

## Conflict of interest

The authors declare that the research was conducted in the absence of any commercial or financial relationships that could be construed as a potential conflict of interest.

## Publisher’s note

All claims expressed in this article are solely those of the authors and do not necessarily represent those of their affiliated organizations, or those of the publisher, the editors and the reviewers. Any product that may be evaluated in this article, or claim that may be made by its manufacturer, is not guaranteed or endorsed by the publisher.
